# 

**Published:** 1874-10

**Authors:** 


					﻿TERMS, ALWAYS IN ADVANCE: One copy, per annum.... $3 00
Yearly subscriptions must begin with the January number.
Persons who send subscriptions for the Journal will please consider
the first number received after subscription as a su fficient receipt for money sent.
2^" We cannot return rejected manuscripts.
Back Nos. for 1872, ’73 and ’74 can be supplied at 30 cents each.
				

